# Insulinaemic potential of diet and lifestyle and risk of type 2 diabetes in the Iranian adults: result from Yazd health study

**DOI:** 10.1186/s12902-023-01364-9

**Published:** 2023-07-03

**Authors:** Morteza Omrani, Mahdieh Hosseinzadeh, Sakineh Shab Bidar, Masoud Mirzaei, Farshad Teymoori, Azadeh Nadjarzadeh, Fatemeh Dehghani Firouzabadi, Seyedeh Tayebeh Rahideh

**Affiliations:** 1 Department of Nutrition, School of Public Health, Iran University of Medical Sciences, Tehran, Iran; 2 Nutrition and Food Security Research Center, School of Public Health, Shahid Sadoughi University of Medical Sciences, Yazd, Iran; 3 Department of Nutrition, School of Public Health, Shahid Sadoughi University of Medical Sciences, Yazd, Iran; 4 Department of Community Nutrition, School of Nutritional Sciences and Dietetics, Tehran University of Medical Sciences, Tehran, Iran; 5 Yazd Cardiovascular Research Center, Yazd Non-communicable Disease Research Centre, Shahid Sadoughi University of Medical Sciences, Yazd, Iran; 6 Nutrition and Endocrine Research Center, Shahid Beheshti University of Medical Sciences, Tehran, Iran

**Keywords:** Empirical dietary index for hyperinsulinaemia, Empirical dietary indices for lifestyle, Empirical dietary index for insulin resistance, Empirical lifestyle index for insulin resistance, Type 2 diabetes

## Abstract

**Background:**

Previous studies have shown that insulin directly affects the risk of type 2 diabetes mellitus (T2DM) but the relationship between insulinaemic potential of diet and lifestyle and the T2DM risk is still unknown. Accordingly, we aimed to investigate the relationship between the insulinaemic potential of diet and lifestyle based on indices including empirical dietary index for hyperinsulinaemia (EDIH), empirical lifestyle index for hyperinsulinaemia (ELIH), empirical dietary index for insulin resistance (EDIR) and empirical lifestyle index for insulin resistance (ELIR) and the T2DM risk in the Iranian adults.

**Methods:**

This study was performed on data of enrollment phase of the Yazd Health Study (YaHS) and TAghzieh Mardom-e-Yazd (Yazd Nutrition Study) (TaMYZ) on 5714 adults aged 20–70 years (mean: 36.29 years). A validated food frequency questionnaire and clinical tests were used to assess food intake and T2DM ascertainment, respectively. We used the Cox regression analysis for determining the relationship between the indices and T2DM risk.

**Results:**

After adjusting for confounding variables, our findings showed that diet with higher ELIH score is 2.28 times more likely for T2DM risk (RR 2.28 [95% CI 1.69–2.56]), but there was no significant relationship between the EDIH, ELIR and EDIR scores and T2DM risk in adults, in the entire study population.

**Conclusions:**

Our findings suggest that diets with higher ELIH score increases the T2DM risk, but there was no significant relationship between the EDIH, ELIR and EDIR scores and T2DM risk. Further epidemiological studies are needed to confirm our findings.

**Supplementary Information:**

The online version contains supplementary material available at 10.1186/s12902-023-01364-9.

## Background

Type 2 diabetes mellitus (T2DM) is a general term for different metabolic disorders, the main consequence of which is chronic hyperglycemia and it can be caused by impaired insulin secretion or impaired insulin function or both [1]. Worldwide, about 462 million people have T2DM, equivalent to 6.28% of the world’s population, and this number is increasing rapidly and is projected to reach 10.2% (578 million) by 2030 and reaching 10.9% (700 million) by 2045 [2, 3]. It is estimated that the annual prevalence of T2DM in Iran is 1% and 85.5% of diabetic patients in Iran have T2DM [3, 4]. T2DM is a costly disease in the Iranian healthcare system, accounting for more than 8.69% of total treatment costs [5].

Several pathogenic processes are involved in the progression of T2DM, including autoimmune destruction of pancreatic cells due to persistent insulin deficiency and disorders of carbohydrate, fat, and protein metabolism that lead to insulin resistance [6]. In general, lifestyle risk factors associated with hyperinsulinemia and T2DM include genetics, diet, physical inactivity, smoking, coffee, tea, alcohol consumption, duration and quality of sleep, depression and stress, and socio-economic status, of which overweight or obesity and physical inactivity are more important because they are the main determinants of insulin resistance and hyperinsulinemia [7–9]. Studies have shown that insulin directly affects the risk of T2DM by regulating energy and glucose metabolism [10]. In addition, C-peptide concentration is considered as a valid marker of hyperinsulinemia and it can predict the prevalence of T2DM; on the other hand, given that hyperinsulinemia is an important risk factor for T2DM [11, 12].

Considering the interaction of different foods with each other and the cumulative effect of diet on the body’s homeostasis, according to previous studies recently, four new dietary and lifestyle indices including the empirical dietary index for hyperinsulinaemia (EDIH) and the empirical dietary indices for hyperinsulinaemia (ELIH), the empirical dietary index for insulin resistance (EDIR), and the empirical lifestyle index for insulin resistance (ELIR) in connection with the possibility of developing chronic non-communicable diseases including some cancers, obesity and insulin resistance have been emerged by health researchers [13–16]. These indices consist of direct and indirect dietary and lifestyle components; the empirical indices for hyperinsulinaemia (EDIH and ELIH) can predict plasma C peptide as a long-term marker of endogenous insulin and the empirical indices for insulin resistance (EDIR and ELIR) based on increasing the triglyceride/ high-density lipoprotein-cholesterol (TG/HDL-c) ratio, they evaluate insulin resistance [12, 17]. Following these studies, the results of the study by Lee et al. in 2020 showed the individuals in highest EDIH quintiles had 2.34 times higher T2DM risk compared with those in lowest quintiles [18]. Also, in a study by Jin et al., conducted in 2021 in postmenopausal women, it was concluded that participants who consumed food patterns with the highest EDIH score, 1.49 times compared to the lowest quintile, had a higher risk of T2DM [19]. However, in the study of Farhadnejad et al., which was conducted in 2021 with the aim of investigating the relationship between EDIH, ELIH, EDIR and ELIR with the risk of T2DM, it was shown that there was no significant relationship between the EDIH score and the risk of T2DM even after adjusting for possible confounders [20].

Given that the results of previous studies, especially for the EDIH, are contradictory and different. In addition, in relation to other indices, the results are positive but limited to one study and because food habits are different in different regions, we hypothesized that insulinaemic potential of diet and lifestyle based on indices including EDIH, ELIH, EDIR and ELIR may be related to the T2DM risk. Therefore, we decided to investigate relationship between insulinaemic potential of diet and lifestyle based on the afore-mentioned indices and T2DM risk in the Iranian adults.

## Materials and methods

### Study design and population

The present study is performed in the framework of Yazd health study (YaHS) and TAghzieh Mardom-e-Yazd (Yazd Nutrition Study) (TaMYZ) study.

The YaHS is a prospective study that has examined the health status and chronic non-communicable diseases (NCDs) and related risk factors among 20–70 year adults of the residents of Yazd, Iran. Study design, sample selection, characteristics of participants in the study, as well as details on data collection methods have been published elsewhere [21]. Data collection was done in two main phases among the participants. In the first phase in November 2014, trained interviewers collected detailed information on personal and dietary habits, physical activity, medical history, mental health status and social well-being of the participants, as well as their anthropometric measurements, and biochemical data. In the second phase of the study in November 2015, the Yazd biobank (Zist Bank-e-Yazd-ZIBA) and a nutrition study named TAMYZ study was established for measurements of genetic and nutritional data.

The inclusion criterion for the current study was completion of relevant questionnaires during two implementation phases. Also, exclusion criteria include suffering form cardiovascular diseases (CVDs), cancer, diabetes, pregnancy, and under- or over-reported dietary energy intakes (out of the range 500–5000 kcal/d) or not responding at least ≥ 20% of items of the dietary questionnaire [21].

**Fig. 1:**
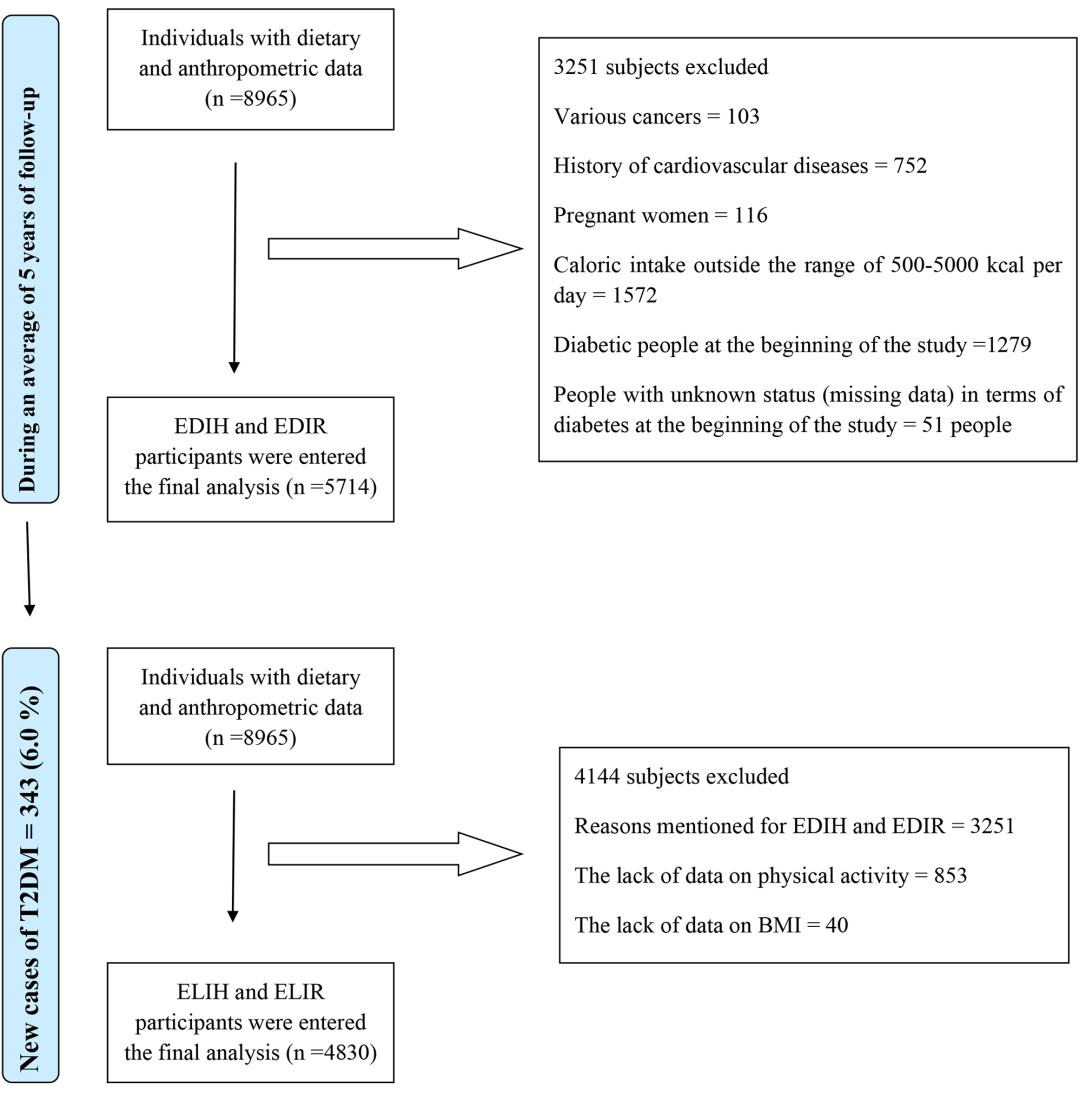
Flow chart of the Yazd Health study (YaHS) participants. (BMI, body mass index; EDIH, empirical dietary index for hyperinsulinaemia; ELIH, empirical dietary indices for lifestyle; EDIR, empirical dietary index for insulinresistance; ELIR, empirical lifestyle index for insulin resistance)

For the present study, of 8965 individuals with complete dietary and anthropometric data, after excluding participants with history of CVDs (n = 752), prevalent cancer (n = 103), pregnant women (n = 116), those with under- or over-reported dietary energy intakes (n = 1572), participants with diabetes in the baseline (n = 1279), and those with missing data of diabetes (n = 51), 5714 participants entered into study for follow-up. As all participants were followed for diabetes incident, all of them remained for final analysis. Regarding the excluding of missing data on physical activity (n = 853) and BMI (n = 40) that are necessary for calculating the ELIR and ELIH, 4830 participants were entered into study for assessing the ELIR and ELIH relationship with diabetes incidence and all of them were followed and remained for final analysis (Fig. 1).

All the experimental protocols were conducted in accordance with the guidelines of the declaration of Helsinki. In addition, all the subjects before participating in the study signed informed consent [21]. This study was approved by the research ethics committee of Iran University of Medical Sciences Faculty of Health with the code of ethics (IR.IUMS.REC.1400.124).

### Anthropometric and biochemical assessments

Body weight was measured with a portable digital scale (Omron BF511 Inc. Nagoya, Japan) with an accuracy of 0.1 kg. All anthropometric measurements were calculated with three repetitions: before the interview begins and after completing one-third and two-thirds of the questionnaire. Height was measured in the standing position by the use of a tape measure on a straight wall with an accuracy of 0.1 cm. Body mass index (BMI) (kg/m^2^) was calculated by weight and height measurements according to the following formula: weight (kg)/height squared (m^2^) [21].

Laboratory measurements including fasting blood glucose (FBG) (mg/dl), TG, low-density lipoprotein-cholesterol (LDL-c), HDL-c and total serum cholesterol were conducted in the YaHS-TAMYZ cohort study according to the standard laboratory protocol using Pars Azmoon kits and calibrated Ciba Corning (Ciba Corp, Basle, Switzerland) auto-analyzers [21].

### Physical activity measurement

All individual physical activity during the week is measured by International Physical Activity Questionnaire (IPAQ) through face-to-face interviews [22]. Then it was converted to the metabolic equivalent per week (MET-h/wk) and sorted to sedentary, moderate and active, based on the median of the MET-h/week levels [22]. The validity and reliability of this FFQ has been assessed in Iran previously [23].

### Assessment of other covariates

The required information regarding age, marriage, level of education, employment status, residence status, immigration status, type of insurance, religion status of individuals, number of children was obtained using a general information questionnaire. Other information on mortality, cancer prevalence, surgery, ischemic heart disease and stroke was obtained from the Electronic Health Record System (SEPAS) using results recorded in public and private hospitals information [21].

Socio economic status variable (SES) using 4 variables including housing status (having a residential house (1 point), not having a residential house (0 points)), occupation status (employed (1 point), unemployed (0 points)), literacy (diploma and above (1 point), below diploma (0 points)) and the number of family members (less than 4 people (1 point), 4 people and more (0 points)) were established. Then, the total points of each variable were added together and a number between 0 and 4 points was obtained for each person. According to the frequency of scores in the studied population, people with scores of 0, 1, and 2 were divided as poor status, people with score 3 as average status, and people with score 4 as good socioeconomic status [24].

### Dietary assessment

Dietary intakes in the YaHS-TAMYZ study were assessed using a 178-item validated, multiple-choice semi-quantitative FFQ [25]. The validity and reliability of the questionnaire has been assessed in Iran previously [26, 27]. In this questionnaire, participants were asked by trained interviewers to report their usual consumption frequency of food items in the last12 months by answering 10-multiple-choice frequency responses ranging from “never or less than once a month” to “10 or more times per day”. In addition, FFQ had five choices for portion size for estimation of the amount of each consumed food [26]. All foods were converted to grams/day based on household portion size of food intakes [28]. The Iranian food composition table (FCT), and U.S. Department of Agriculture FCT for the foods that were not available in the Iranian FCT were used to calculate energy and nutrient intakes per each gram of any food item [29].

### T2DM ascertainment

Diabetes diagnosis was ascertained based on standard method (about 40% of participants using laboratory measurements) and verbal autopsy (self-reported) at the baseline of study [21, 30]. However, incidence of diabetes extracted from an aggregated electronic database which was obtained from the Electronic Health Record System (SEPAS) using results recorded in public and private hospitals information of the Yazd [21].

### Calculation of indices

EDIH is calculated based on two groups of food components including positive and negative determinants, which are direct and positive determinants including red meat, processed meat, cream soup, margarine, low-energy drinks, poultry, high-energy drinks, butter, French fries, low-fat dairy products, eggs, tomatoes and other fish. The negative and inverse determinants of this food index included coffee, high-fat dairy products, and green leafy vegetables, red wine and whole fruits [12, 17]. Each of the above foods is multiplied by a particular weight previously calculated in the study conducted by Tabung and his colleague [17]. Finally, all of the foods are added together as a given weight and the totals score was calculated for each person. ELIH score is based on a set of direct and inverse components and the positive and direct components of this index include body mass index (BMI), liquor, margarine, cream soup, butter, fruit juice and red meat. Negative and inverse components also include whole fruit, coffee, red wine, physical activity, high-fat dairy products, snacks and salad dressings [12, 17]. Similar to the calculation of EDIH mentioned above, ELIH is also calculated.

EDIR score is determined based on a set of direct and indirect components and the direct components of this index include low energy drinks, margarine, refined grains, tomatoes, other fish, fruit juice, red meat, processed meat, cream soup and other vegetables. Negative and indirect components also include green leafy vegetables, coffee, high-fat dairy, yellow vegetables and nuts [12, 17]. Also, ELIR is determined based on a set of direct and inverse components. The positive and direct components of this index include BMI, low energy drink, tea, margarine, refined grains, tomatoes, potatoes, other vegetables, fruit juices, processed meats and red meats. Negative and inverse components also include coffee, red wine, liquor, green leafy vegetables, high-fat dairy products and physical activity [12, 17]. Similar to the calculation of EDIH and ELIH, each of the above foods is multiplied by the food statistical weight and finally all of the food scores are summed together as a given weight and EDIR and ELIR scores were calculated for each person.

Note that due to regional sensitivities, information on the amount of alcohol consumption was not collected. Alcoholic beverages include red wine, liquor, and beer; also, two other items and groups of index components, including cream soup and low-energy drink, were not included in the YaHS questionnaire and Iranian people eating habits [21]. Overall, in the case of the EDIH, 15 food groups out of 18 items were used in calculating the score of this index. In the case of the ELIH, 11 of the 14 main components of this index were used, and in the EDIR, 13 items out of 18 and ELIR, 13 out of 17 food groups. The food items in these four indices were used and replaced based on the 39 food groups defined in the YaHS FFQ [21, 31].

### Statistical methods

Statistical analyses of data were done using SPSS20.0 software (version 20.0; SPSS Inc, Chicago IL). The normality of the distribution of variables was evaluated using histogram, p-p plot, and Kolmogorov-Smirnov test. To remove the strong effect of energy intake on the magnitude of dietary indices including EDIR and EDIH, these scores were calculated per 1000 kcal of energy intake for each participant. Then, for assessing the association between insulinemic indices with the risk of diabetes, study population were divided into three groups based on each of these dietary or lifestyle indices. Baseline characteristics of the study participants were presented among diabetic and non-diabetic people and across the tertiles of the afore-mentioned indices. The data were reported as mean ± standard deviation or median and inter quartile range of 25–75 for quantitative variables and percentage for qualitative variables. Comparison of averages between diabetic and non-diabetic subjects was conducted using independent sample t-test and chi-square test for continuous and categorical variables, respectively. In addition, to test the trend of qualitative and quantitative variables across tertiles of insulinemic indices (as median value in each tertile), Chi-square and linear regression were used, respectively.

Cox regression analysis was used to investigate the relationship between dietary and lifestyle insulin indices with the risk of T2DM, and the relative risk (RR) and 95% CI were reported. P-value less than 0.05 was considered as statistically significant values.

## Results

The mean age ± standard deviations (SD) of age and BMI among all participants (51% male) were 36 ± 7.8 years and 26.71 ± 5.21 kg/m², respectively. Also, the mean ± SD of EDIH, ELIH, EDIR and ELIR scores were 0.24 ± 0.28, 1.51 ± 0.45, 0.32 ± 0.17, and 4.83 ± 2.54, respectively. Baseline characteristics of the study participants based on the T2DM are presented in Table 1. People diagnosed with diabetes compared to people who were not diagnosed with diabetes, were older, and had higher BMI, family history of diabetes, and included menopausal women, married people, house owners and fiber intake. In addition, these people had significantly less academic education, physical activity and number of men. Among the insulin indices, the ELIH and EDIR in diabetic patients compared to not-diabetic patients had a significant higher and lower mean score, respectively but in other indices (EDIH and ELIR), there wasn’t any significance.

**Table 1:** Study population characteristics based on diabetic and non-diabetic subjects at the end of the second phase of the Yazd Health Study

	non-diabetic (n = 5371)	diabetic (n = 343)	P value
**Demographic data**			
Age (year)			< 0.001
20–29 years (%)	24.7	5.6	
30–39 years (%)	23.9	9.7	
40–49 years (%)	22.7	27.6	
50–59 years (%)	16.1	32.1	
60–69 years (%)	12.6	25.0	
Male (%)	51.4	45.1	0.024
Body mass index (Kg.m^2^)	26.5 ± 5.1	29.8 ± 4.9	< 0.001
Physical activity (MET/min/week)	17.8 ± 15.3	14.4 ± 12.4	< 0.001
Smoking (yes, %)	11.0	11.7	0.708
Menopausal status (yes, %)	13.4	31.2	< 0.001
Marital status (married, %)	83.3	90.6	< 0.001
Education level (diploma and higher, %)	51.7	33.8	< 0.001
Family size (≤ 4 member, %)	73.7	71.5	0.380
House acquisition (yes, %)	76.6	88.2	< 0.001
Occupation status (employed, %)	82.6	81.2	0.530
Socio economic status (%)			0.060
Low (%)	31.4	34.0	
Middle (%)	45.2	48.4	
High (%)	23.4	17.6	
Family history of diabetes (%)	34.9	45.5	< 0.001
**Dietary intake**			
Energy intake (Kcal/d)	2574 ± 973	2573 ± 1012	0.991
Carbohydrate (% of energy)	53.1 ± 7.8	53.8 ± 7.7	0.128
Protein (% of energy)	15.5 ± 3.9	15.2 ± 3.8	0.082
Fat (% of energy)	31.3 ± 6.9	31.0 ± 6.6	0.444
Polyunsaturated fatty acids (% of energy)	8.1 ± 4.9	7.8 ± 3.7	0.202
Fiber (g/1000 Kcal)	9.1 ± 4.2	9.7 ± 4.0	0.006
**Insulin Indices**			
EDIR (per 1000 Kcal)	0.32 ± 0.18	0.30 ± 0.15	0.036
EDIH (per 1000 Kcal)	0.08 (0.04–0.133)	0.068 (0.028–0.125)	0.070
ELIR	4.83 ± 2.55	4.75 ± 2.38	0.573
ELIH	1.50 ± 0.45	1.67 ± 0.48	< 0.001

Data represented as mean ± standard deviation, or median (IQR 25–75) for continues variables and number and percent for categorical variables

*The comparison of means between diabetic and non-diabetic people was done using independent t-test and chi-square test for quantitative and qualitative variables, respectively

EDIH, empirical dietary index for hyperinsulinemia; ELIH, empirical lifestyle index for hyperinsulinemia; EDIR, empirical dietary index for insulin resistance; ELIR, empirical lifestyle index for insulin resistance,

Regarding the baseline values of different variables and literature review of previous studies, we selected some potential confounders and evaluated the insulin indices and diabetes incidence relationship using two adjusted models:1-age and sex adjusted model, 2- final model adjusted for BMI (only for food indices EDIR and EDIH), physical activity (only for EDIR and EDIH dietary indices), smoking, family history of diabetes, marital status, socioeconomic status, menopause status and daily energy intake.

The results of cox regression analysis of four insulinemic indices and incidence of diabetes are shown in Table 2. Higher adherence of EDIR, EDIH, and ELIR were not significantly associated with the risk of diabetes incidence in different statistical models. After adjusting for age, sex, smoking, family history of diabetes, marital status, socio economic status, menopausal status and dietary intake of energy; the RR (95% CI) of diabetes incidence were 0.95 (0.70–1.28), 0.77 (0.58–1.02), and 0.95 (0.65–1.37) for participants who were in the highest versus lowest tertiles of EDIR, EDIH, and ELIR, respectively. However, higher adherence to ELIH score was significantly associated with higher risk of incidence of diabetes in all models of Cox regression. The RR (95% CI) of diabetes incidence in participants in the highest vs. lowest tertiles of ELIH were 2.47 (1.73–3.53) and 2.28 (1.59–3.27) in the age and sex adjusted model and final adjusted model, respectively.

**Table 2:** Relative risk (RR) (95% CI) of T2DM according to insulinaemic potential of diet and lifestyle indices tertiles (result from Yazd Health Study)

	Relative risk of diabetes (95% CI)			P for trend
	T1	T2	T3	
**EDIR (1000 Kcal)**				
Median score	0.199	0.300	0.431	
Case/Total	127 / 1905	110 / 1903	106 / 1906	
Crude model	1.00 (Ref)	0.87 (0.66–1.15)	0.83 (0.62–1.10)	0.188
Model 1*	1.00 (Ref)	0.90 (0.69–1.22)	0.92 (0.69–1.22)	0.571
Model 2^†^	1.00 (Ref)	0.88 (0.66–1.19)	0.95 (0.70–1.28)	0.773
**EDIH (1000 Kcal)**				
Median score	0.023	0.080	0.156	
Case/Total	133 / 1904	110 / 1905	100 / 1905	
Crude model	1.00 (Ref)	0.78 (0.60–1.03)	0.70 (0.53–0.94)	0.018
Model 1^*^	1.00 (Ref)	0.83 (0.63–1.10)	0.76 (0.57–1.01)	0.060
Model 2^†^	1.00 (Ref)	0.83 (0.63–1.09)	0.77 (0.58–1.02)	0.079
**ELIR**				
Median score	2.95	3.43	6.18	
Case/Total	102/ 1609	101 / 1611	91 / 1610	
Crude model	1.00 (Ref)	0.98 (0.72–1.33)	0.93 (0.68–1.27)	0.647
Model 1	1.00 (Ref)	1.15 (0.84–1.58)	1.08 (0.78–1.49)	0.838
Model 2^‡^	1.00 (Ref)	1.11 (0.80–1.53)	0.95 (0.65–1.37)	0.572
**ELIH**				
Median score	1.14	1.45	1.83	
Case/Total	52/ 1610	93 / 1609	149 / 1611	
Crude model	1.00 (Ref)	2.03 (1.39–2.95)	3.14 (2.21–4.47)	< 0.001
Model 1^*^	1.00 (Ref)	1.75 (1.20–2.55)	2.47 (1.73–3.53)	< 0.001
Model 2^‡^	1.00 (Ref)	1.70 (1.17–2.48)	2.28 (1.59–3.27)	< 0.001

* Obtained by Cox regression analysis. Crude: no adjustments

^*^Model 1: adjusted for age and sex

^†^ Model 2: adjusted for model 1 and body mass index, smoking, physical activity, family history of diabetes, marital status, socio economic status, menopausal status and dietary intake of energy

EDIH, empirical dietary index for hyperinsulinemia; ELIH, empirical lifestyle index for hyperinsulinemia; EDIR, empirical dietary index for insulin resistance; ELIR, empirical lifestyle index for insulin resistance

The basic characteristics of the study participants according to the EDIR score are presented in **Supplementary Table 1**. The participants in the highest tertile compared to the people in the lower tertile had significantly higher academic education, were older and had higher protein consumption. Also, in these people, the number of family members (4 people and less, %), the number of menopausal women, energy intake, fat intake, polyunsaturated fatty acids (PUFA) and fiber intake were lower. Regarding the food groups, the consumption of margarine, dark yellow vegetables, processed meats, red meat, other fish, other vegetables, tomatoes, refined grains was significantly higher. In addition, the intake of coffee, green leafy vegetables, nuts and high-fat dairy products were significantly lower but no significant relationship was observed between the study subjects regarding other variables.

The basic characteristics of the study participants according to the EDIR score are presented in **Supplementary Table 1**. The participants in the highest tertile had significantly higher academic education, older and had higher protein consumption. Also, in these people, the number of family members (4 people and less, %), the number of menopausal women, energy intake, fat intake, polyunsaturated fatty acids (PUFA) and fiber intake were lower. Regarding the food groups, the consumption of margarine, dark yellow vegetables, processed meats, red meat, other fish, other vegetables, tomatoes, refined grains was significantly higher. In addition, the intake of coffee, green leafy vegetables, nuts and high-fat dairy products were significantly lower but no significant relationship was observed between the study subjects regarding other variables.

The basic characteristics of the study participants according to the EDIH score are presented in **Supplementary Table 2**. The participants in the highest tertile had significantly higher physical activity, academic education, the number of men, energy intake, protein intake, fat intake and PUFA (% energy) intake. In contrast, age, number of family members (4 people and less, %), menopausal women, carbohydrate and fiber (g/1000 kcal) intake were significantly lower. About the food groups, the consumption of margarine, butter, poultry, processed meats, red meats, French fries, other fish, high energy beverages, tomatoes, low-fat dairy products and eggs were significantly higher in these people and the intake of high-fat dairy products, whole fruit, green leafy vegetables, and the intake of coffee were less. Regarding other variables, no significant relationship was observed among the study subjects. Baseline characteristics of the study participants based on the ELIR score are presented in **Supplementary Table 3**. The participants in the highest tertile compared to the people in the lower tertile were younger, had significantly higher BMI, academic education, energy and fat consumption, while the number of postmenopausal women and protein intake level was significantly lower. About the food groups, intake of margarine, processed meats, red meat, potatoes, other fish, refined grains, tomatoes, other vegetables, fruit juice and green leafy vegetables were significantly more in these people and coffee intake level was significantly lower. Regarding other variables, no significant relationship was observed between the studied subjects.

Baseline characteristics of the study participants according to the ELIH score are presented in **Supplementary Table 4**. The participants in the highest tertile had significantly higher BMI, the academic education, the number of employed people, married people, house owners, and postmenopausal women, number of people with family history of diabetes, consumption of energy, protein and fat intake. While physical activity, smokers and the number of men, carbohydrate and fiber intake were significantly lower. About the food groups, intake of margarine, butter, red meats and the intake of high-fat dairy products were significantly more in these people and the intake of snacks, fruit juice, salad dressing and the intake of coffee was significantly lower. Regarding other variables, no significant relationship was observed between the studied subjects.

## Discussion

The results of the present study showed that people who received a diet with a higher score on ELIH, have a 2.28 times higher risk of T2DM while there was no significant relationship between the risk of T2DM and the scores of EDIH, ELIR and EDIR indices after adjusting the effect of confounding factors including age, sex, BMI, smoking, physical activity, family history of diabetes, marital status, socio economic status, menopausal status and dietary intake of energy in the entire study population.

According to our knowledge, three studies have been conducted to investigate the relationship between EDIH, ELIH, ELIR and EDIR indices with the risk of T2DM, however, these studies were carried out in different populations and different regions [18–20]. But, the association of other dietary insulinemic indices such as glycemic index (GI), glycemic load (GL), insulin index (II) and insulin load (IL) has been assessed with the risk of T2DM [32, 33].

In the study by van Dam et al., they concluded that a diet with a high GI and GL is not negatively associated with metabolic risk factors (total blood cholesterol, HDL cholesterol, triglycerides or insulin or glucose (fasting or post-loaded)) [32]. Although GI and GL assess postprandial glycemic potential based on dietary carbohydrate content, indices of insulinemic potential and insulin resistance assess plasma insulin response regardless of macronutrient content [12, 17, 34]. Another study indicated that II and IL do not have a significant relationship with HbA1c and C-peptide [33]. It should be noted that the insulin indices are fundamentally different from the insulin potential indices; first, the insulin and insulin resistance potential indices use food groups instead of individual foods to measure the index score. Secondly, the insulin indices do not establish a relationship with endogenous and long-term insulin, in contrast to the insulin potential indices [17, 35].

Farhadnejad et al. study like our research showed no significant relationship between the EDIH score and the risk of T2DM. They justified the results of this study, with the reasons such as the low intake of EDIH components and the diversity of the studied population [20]. However, in another study conducted by Lee et al., using three U.S. (United States) cohort data, they concluded that after adjusting for the BMI in higher quintiles of EDIH, compared to those in lower quintiles, 2.34 times more individuals were at the risk of T2DM [18]. In our study, compared to previous studies, people generally had less and different food intake in the main components of the indices, which can be effective in the final score of the indices and in the results of measuring the relationship of these indices with the risk of T2DM; for example, the average intake of red meat, processed meat and dairy products, which are the main components of most of these indices, in Yazd adult population, was much lower than Lee study [18]. Hence the differences in food pattern and economic issues of the two countries could have been shown as reasons. In addition, differences in the adjustment of possible confounders in different studies may also lead to the differences in the findings [18]. Another study conducted by Jin et al., in postmenopausal women indicated that participants with the highest EDIH score, 1.49 times compared to the lowest quintile, had a higher risk of developing T2DM [19]. Since his study was conducted on postmenopausal women, it may not be possible to compare the results of the two studies.

Previous study revealed association of EDIH with the risk of weight gain that could indicate the possibility of BMI and obesity mediation in positive results regarding the risk of T2DM [14]. In relation to this case, in the study of Farhadnejad et al., after adjusting BMI, the EDIH and ELIR remained meaningless [20]. In contrast, in the other two studies of Jin et al. and Lee et al., although the results remained significant after BMI adjustment, the intensity of the relationship with the risk of T2DM was weakened [18, 19]. Also, we know T2DM risk decreases with increasing physical activity; moreover, physical activity as one of the differential components of ELIH compared to other indices in this study has played an important role in making the relationship meaningful [36]. The afore-mentioned cases show the important role of BMI and physical activity in the risk of T2DM, independently.

Other important factors in the non-significance of the relationship between the three mentioned indices and the risk of T2DM can be the heterogeneity and inequality of food groups intake; For example, in our study, intake of high-fat dairy products as one of the negative components of EDIH and intake of low-fat dairy products as one of the positive components of EDIH was generally low, which may be due to ignoring the type of dairy consumed (high-fat or low-fat) in Iranian people [17, 37]. while the per capita consumption of dairy products in Iran in 2014 was 60 kg which is very low and it is only half of the global average [37]. Also, coffee consumption as one of the negative components of the EDIH and margarine consumption as one of the positive components of the EDIH was very low in our study, which indicates the less significant role of coffee and margarine consumption in the daily eating habits of Iranian people [17, 38]. Besides, per capita consumption of coffee in Iran per day is much less compared to the U.S and coffee consumption in Iran is mostly industrialized and sweetened, which disrupts the main effect of coffee in the diet [38]. As mentioned in the method section, due to regional sensitivities, information on the amount of red wine, liquor, and beer consumption as components of indices and two other groups, including cream soup and low-energy drink, which is lacking in the YaHS questionnaire and eating habits of Iranian people have not been collected [21]. These matters probably could affect the final score of indices and could be another reason for absence of the relationship between the three mentioned indices and the risk of T2DM in our study.

The possible mechanisms for the positive association between ELIH score and the risk of T2DM in different communities is explained by the fact that people with a higher ELIH score actually had a higher probability of producing C-peptide as a marker of endogenous and long-term insulin production, which is associated with a higher risk of T2DM [12, 17]. Also, BMI is a direct and positive part of this index that can express the concept of the mediating effect of obesity and risk of T2DM [12, 17]. moreover, most of the positive components of this index (margarine, cream soup, butter, fruit juice, red meat) have a higher energy density that has been associated with obesity and T2DM [12, 17].

Our study also had some limitations as follows: First, as mentioned earlier in our study, information about alcoholic food groups and two other groups have not been collected that also could affect the results. Second, despite the fact that we used semi quantitative FFQs to bring together dietary intake data with skilled interviewers, there is a possibility of measurement error and misclassification. Third, although we tried to fully adjust potential important confounder, we cannot completely rule out the possibility of the confounders by unmeasured variables. Fourth, in some part of the studied population, the measurement of T2DM data has been done in the form of self-report, which could have effects on our findings. In addition, since the study participants were residents of Yazd Greater Area, but the generalization of the findings should be considered cautiously and more research is needed to replicate our findings in different racial and ethnic groups. On the other hand, according to our knowledge, this study is the first study that investigated the relationship between EDIH, ELIH, ELIR and EDIR and risk of T2DM in adults in Yazd, which can be considered as one of the strengths of the study. Furthermore, the high sample size and the control of a wide range of possible confounders in the experiment and analysis are other strengths of this study.

## Conclusions

Our findings showed that people who received a diet with a higher score on the ELIH, after adjusting for confounders, they have a 2.28 times higher risk of T2DM while there was no significant relationship between the risk of T2DM and the scores of EDIH, ELIR and EDIR indices in adults of Yazd Greater Area. Due to the lack of exact knowledge of the underlying mechanism of the relationship between insulin potential indices and insulin resistance with T2DM, more molecular and genetic studies are needed by taking into account different clinical factors.

## Electronic supplementary material

Below is the link to the electronic supplementary material.

Supplementary Material 1

## Data Availability

The datasets used and/or analyzed during the current study are available from the corresponding author on reasonable request.

## Abbreviations

BMI: body mass index
EDIH: empirical dietary index for hyperinsulinaemia
ELIH: empirical dietary indices for lifestyle
EDIR: empirical dietary index for insulin resistance
ELIR: empirical lifestyle index for insulin resistance
FFQ: food frequency questionnaire
FBG: fasting blood glucose
TG: triglycerides
LDL-c: low-density lipoprotein-cholesterol
HDL-C: high-density lipoprotein cholesterol
RR: relative risk
SD: standard deviation
T2DM: type 2 diabetes mellitus
TaMYZ: TAghzieh Mardom-e-Yazd (Yazd Nutrition Study)
TG: triglyceride
YaHS: Yazd Health Study
