# Supplementary material for: Insulinaemic potential of diet and lifestyle and risk of type 2 diabetes in the Iranian adults: result from Yazd health study
Source: BMC Endocr Disord. 2023 Jul 3;23:136. doi: 10.1186/s12902-023-01364-9 (PMC10316619; doi:10.1186/s12902-023-01364-9)
Supplement: Supplementary file 1 — Supplementary Material 1 [file 12902_2023_1364_MOESM1_ESM.docx]

**Supplementary Table 1:** Study population characteristics based on the tertiles of EDIR score (per 1000 Kcal) among the Yazd Health Study

|  | T1 (n=1905) | | T2 (n=1903) | T3 (n=1906) | P for trend |
| --- | --- | --- | --- | --- | --- |
| **Demographic data** |  | |  |  |  |
| Age (year) |  | |  |  | <0.001 |
| 20-29 years (%) | 21.2 | | 23.3 | 26.1 |  |
| 30-39 years (%) | 23.6 | | 21.5 | 24.1 |  |
| 40-49 years (%) | 22.1 | | 24.5 | 22.5 |  |
| 50-59 years (%) | 17.9 | | 16.9 | 16.2 |  |
| 60-69 years (%) | 15.2 | | 13.7 | 11.2 |  |
| Male (%) | 49.7 | | 51.2 | 52.2 | 0.299 |
| Body mass index (Kg.m^2^) | 26.8±5.1 | | 26.7±5.4 | 26.5±4.9 | 0.089 |
| Physical activity (MET/hour/week) | 17.7±15.3 | | 17.0±15.1 | 18.1±15.2 | 0.135 |
| Smoking (yes, %) | 11.2 | | 11.0 | 10.9 | 0.970 |
| Menopausal status (yes, %) | 17.5 | | 14.1 | 11.8 | <0.001 |
| Marital status (married, %) | 83.8 | | 84.5 | 82.9 | 0.409 |
| Education level (diploma and higher, %) | 47.8 | | 50.3 | 53.7 | 0.002 |
| Family size (≤ 4 member, %) | 75.2 | | 71.4 | 74.0 | 0.028 |
| House acquisition (yes, %) | 77.2 | | 77.6 | 77.1 | 0.909 |
| Occupation status (employed, %) | 82.6 | | 82.3 | 82.6 | 0.961 |
| Socio economic status (%) |  | |  |  | 0.207 |
| Low (%) | 31.7 | | 32.9 | 30.2 |  |
| Middle (%) | 45.8 | | 44.7 | 45.6 |  |
| High (%) | 22.5 | | 22.4 | 24.2 |  |
| Family history of diabetes (%) | 36.6 | | 35.1 | 34.9 | 0.518 |
| **Dietary intake** |  |  |  |  |  |
| Energy intake (Kcal/d) | 2723±1007 | | 2484±933 | 2514±966 | <0.001 |
| Carbohydrate (% of energy) | 52.8±9.1 | | 53.4±6.7 | 53.2±7.4 | 0.086 |
| Protein (% of energy) | 15.1±4.5 | | 15.8±3.4 | 15.7±3.6 | <0.001 |
| Fat (% of energy) | 32.0±8.2 | | 30.7±5.9 | 31.0±6.3 | <0.001 |
| Polyunsaturated fatty acids (% of energy) | 8.4±5.3 | | 8.1±5.1 | 7.8±3.9 | <0.001 |
| Fiber (g/1000 Kcal) | 9.5±5.4 | | 9.0±3.2 | 8.9±3.4 | <0.001 |
| **Dietary components of EDIR** |  |  |  |  |  |
| Margarine (serving/d) | 0.085 (0.085–0.085) | | 0.085 (0.085–0.335) | 0.085 (0.085–0.335) | <0.001 |
| Red meat (serving/d) | 0.24 (0.10–0.42) | | 0.29 (0.16–0.45) | 0.35 (0.18–0.53) | <0.001 |
| Refined grain (serving/d) | 2.5 (1.8–4.0) | | 3.9 (2.6–4.6) | 4.5 (3.6–7.4) | <0.001 |
| Processed meat (serving/d) | 0.25 (0.25–0.67) | | 0.25 (0.25–0.75) | 0.25 (0.25–0.75) | <0.001 |
| Tomatoes (serving/d) | 0.16 (0.07–0.48) | | 0.48 (0.16–0.57) | 0.48 (0.16–1.13) | <0.001 |
| Other vegetables (serving/d) | 0.99 (0.50–1.46) | | 1.26 (0.81–2.07) | 1.79 (1.10–2.54) | <0.001 |
| Other fish (serving/d) | 0.08 (0.02–0.15) | | 0.08 (0.04–0.16) | 0.06 (0.02–0.16) | <0.001 |
| Fruit juice (serving/d) | 0.14 (0.06–0.43) | | 0.12 (0.06–0.36) | 0.11 (0.06–0.36) | 0.254 |
| Coffee (serving/d) | 0.008 (0.008–0.325) | | 0.008 (0.008–0.325) | 0.008 (0.008–0.325) | <0.001 |
| Green leafy vegetables (serving/d) | 0.29 (0.14–0.66) | | 0.30 (0.15–0.58) | 0.24 (0.12–0.50) | <0.001 |
| High fat dairy (serving/d) | 1.16 (0.85–2.08) | | 1.11 (0.87–1.60) | 1.07 (0.52–1.24) | <0.001 |
| Dark yellow vegetables (serving/d) | 0.12 (0.05–0.35) | | 0.13 (0.06–0.27) | 0.12 (0.06–0.56) | <0.001 |
| Nuts (serving/d) | 0.44 (0.22–0.95) | | 0.44 (0.25–0.83) | 0.36 (0.20–0.56) | <0.001 |

Data represented as mean ±standard deviation (SD), or median (interquartile range (IQR) 25-75) for continues variables and number and percent for categorical variables

* Using ANOVA test for quantitative data and Chi-square test for qualitative data

MET; metabolic equivalent, EDIR; empirical dietary index for insulin resistance

**Supplementary Table 2**: Study population characteristics based on the tertiles of EDIH score (per 1000 Kcal) among the Yazd Health Study

|  | T1 (n=1904) | T2 (n=1905) | T3 (n=1905) | P for trend |
| --- | --- | --- | --- | --- |
| **Demographic data** |  |  |  |  |
| Age (year) |  |  |  | <0.001 |
| 20-29 years (%) | 22.0 | 23.7 | 24.9 |  |
| 30-39 years (%) | 21.9 | 23.1 | 24.1 |  |
| 40-49 years (%) | 22.3 | 24.8 | 22.0 |  |
| 50-59 years (%) | 18.5 | 15.4 | 17.1 |  |
| 60-69 years (%) | 15.2 | 13.0 | 11.9 |  |
| Male (%) | 47.9 | 53.7 | 51.6 | 0.001 |
| Body mass index (Kg.m^2^) | 26.9±5.2 | 26.6±5.2 | 26.6±5.0 | 0.063 |
| Physical activity (MET/hour/week) | 17.0±14.4 | 17.8±15.9 | 17.8±15.2 | 0.026 |
| Smoking (yes, %) | 10.5 | 11.8 | 10.9 | 0.427 |
| Menopausal status (yes, %) | 16.7 | 12.8 | 13.9 | 0.002 |
| Marital status (married, %) | 85.0 | 83.5 | 82.8 | 0.172 |
| Education level (diploma and higher, %) | 48.2 | 50.3 | 53.3 | 0.007 |
| Family size (≤ 4 member, %) | 76.1 | 71.9 | 72.7 | 0.009 |
| House acquisition (yes, %) | 77.4 | 77.9 | 76.7 | 0.663 |
| Occupation status (employed, %) | 82.8 | 82.6 | 82.1 | 0.844 |
| Socio economic status (%) |  |  |  | 0.291 |
| Low (%) | 32.4 | 31.8 | 30.6 |  |
| Middle (%) | 45.2 | 44.8 | 46.1 |  |
| High (%) | 22.4 | 23.5 | 23.2 |  |
| Family history of diabetes, (%) | 36.4 | 35.5 | 34.6 | 0.545 |
| **Dietary intake** |  |  |  |  |
| Energy intake (Kcal/d) | 2506±990 | 2551±946 | 2664 ±982 | <0.001 |
| Carbohydrate (% of energy) | 56.4±7.8 | 53.6±7.8 | 49.3±7.3 | <0.001 |
| Protein (% of energy) | 13.9±3.3 | 15.3±3.0 | 17.4±4.2 | <0.001 |
| Fat (% of energy) | 29.6±8.2 | 31.0±6.5 | 33.1±6.2 | <0.001 |
| Polyunsaturated fatty acids (% of energy) | 7.8±4.9 | 8.2±5.0 | 8.2±4.3 | 0.037 |
| Fiber (g/1000 Kcal) | 10.1±5.6 | 9.0±3.2 | 8.3±2.9 | <0.001 |
| **Dietary components of EDIH** |  |  |  |  |
| Red meat (serving/d) | 0.21 (0.09–0.35) | 0.27 (0.16–0.44) | 0.42 (0.22–0.64) | <0.001 |
| Processed meat (serving/d) | 0.02 (0.02–0.06) | 0.02 (0.02–0.07) | 0.025 (0.02–0.99) | <0.001 |
| Margarine (serving/d) | 0.085 (0.085–0.085) | 0.085 (0.085–0.335) | 0.085 (0.085–0.335) | <0.001 |
| Poultry (serving/d) | 0.18 (0.10–0.27) | 0.27 (0.11–0.52) | 0.36 (0.18–1.07) | <0.001 |
| Butter (serving/d) | 0.085 (0.085–0.167) | 0.085 (0.085–0.335) | 0.167 (0.085–0.335) | <0.001 |
| French fries (serving/d) | 0.015 (0.003–0.023) | 0.015 (0.008–0.032) | 0.019 (0.008–0.049) | <0.001 |
| Other fish (serving/d) | 0.05 (0.021–0.126) | 0.08 (0.049–0.175) | 0.08 (0.03–0.17) | <0.001 |
| High energy beverages (serving/d) | 0.065 (0.016–0.140) | 0.065 (0.016–0.140) | 0.065 (0.016–0.210) | <0.001 |
| Tomatoes (serving/d) | 0.24 (0.07–0.48) | 0.48 (0.16–0.56) | 0.48 (0.16–1.13) | <0.001 |
| Low fat dairy (serving/d) | 0.27 (0.12–0.41) | 0.31 (0.17–0.50) | 0.36 (0.17–0.56) | <0.001 |
| Eggs (serving/d) | 0.21 (0.07–0.45) | 0.30 (0.15–0.90) | 0.45 (0.30–0.90) | <0.001 |
| Coffee (serving/d) | 0.008 (0.008–0.032) | 0.008 (0.008–0.032) | 0.008 (0.008–0.032) | <0.001 |
| Whole fruits (serving/d) | 3.58 (2.78–4.87) | 3.20 (2.62–4.12) | 2.88 (2.46–3.97) | <0.001 |
| High fat dairy (serving/d) | 1.15 (0.86–1.86) | 1.09 (0.80–1.442) | 1.08 (0.57–1.40) | <0.001 |
| Green leafy vegetables (serving/d) | 0.28 (0.14–0.62) | 0.28 (0.14–0.57) | 0.25 (0.12–0.53) | <0.001 |

Data represented as mean ±standard deviation (SD), or median (interquartile range (IQR) 25-75) for continues variables and number and percent for categorical variables

* Using ANOVA test for quantitative data and Chi-square test for qualitative data

MET; metabolic equivalent, EDIH, empirical dietary index for hyperinsulinemia

**Supplementary Table 3**: Study population characteristics based on the tertiles of ELIR score among the Yazd Health Study

|  | T1 (n=1609) | T2 (n=1611) | T3 (n=1610) | P for trend |
| --- | --- | --- | --- | --- |
| **Demographic data** |  |  |  |  |
| Age (year) |  |  |  | <0.001 |
| 20-29 years (%) | 21.3 | 25.0 | 25.1 |  |
| 30-39 years (%) | 21.2 | 24.6 | 25.7 |  |
| 40-49 years (%) | 23.4 | 23.1 | 24.1 |  |
| 50-59 years (%) | 17.6 | 16.5 | 15.7 |  |
| 60-69 years (%) | 16.4 | 10.8 | 9.5 |  |
| Male (%) | 49.8 | 51.7 | 51.4 | 0.512 |
| Body mass index (Kg.m^2^) | 26.2±4.7 | 26.3±5.1 | 27.4±5.6 | <0.001 |
| Physical activity (MET/hour/week) | 17.9±15.6 | 17.3±15.3 | 17.7±14.8 | 0.976 |
| Smoking (yes, %) | 12.0 | 10.0 | 10.4 | 0.167 |
| Menopausal status (yes, %) | 16.8 | 11.9 | 12.4 | <0.001 |
| Marital status (married, %) | 82.8 | 84.2 | 83.8 | 0.544 |
| Education level (diploma and higher, %) | 47.9 | 54.3 | 54.7 | <0.001 |
| Family size (≤ 4 member, %) | 72.9 | 74.6 | 71.4 | 0.126 |
| House acquisition (yes, %) | 77.0 | 78.5 | 76.1 | 0.272 |
| Occupation status (employed, %) | 82.1 | 82.2 | 82.9 | 0.819 |
| Socio economic status (%) |  |  |  | 0.141 |
| Low (%) | 33.6 | 28.9 | 31.4 |  |
| Middle (%) | 43.8 | 47.3 | 44.2 |  |
| High (%) | 22.6 | 23.8 | 24.4 |  |
| Family history of diabetes (%) | 36.9 | 36.2 | 35.8 | 0.540 |
| **Dietary intake** |  |  |  |  |
| Energy intake (Kcal/d) | 2112±798 | 2417±800 | 3227±931 | <0.001 |
| Carbohydrate (% of energy) | 52.3±8.5 | 53.5±7.3 | 53.4±7.6 | 0.006 |
| Protein (% of energy) | 16.2±4.2 | 15.6±3.2 | 14.7±3.9 | <0.001 |
| Fat (% of energy) | 31.4±7.2 | 30.7±6.4 | 31.8±6.9 | 0.004 |
| Polyunsaturated fatty acids (% of energy) | 8.4±5.2 | 8.1±5.1 | 7.7±3.9 | 0.888 |
| Fiber (g/1000 Kcal) | 8.3±5.6 | 7.8±4.8 | 8.1±3.8 | 0.804 |
| **Dietary components of ELIR** |  |  |  |  |
| Red meat (serving/d) | 0.24 (0.12–0.42) | 0.28 (0.16–0.46) | 0.33 (0.15–0.53) | <0.001 |
| Processed meat (serving/d) | 0.025 (0.025–0.032) | 0.025 (0.025–0.067) | 0.025 (0.025–0.111) | <0.001 |
| Margarine (serving/d) | 0.085 (0.085–0.085) | 0.085 (0.085–0.335) | 0.085 (0.085–0.335) | <0.001 |
| Tomatoes (serving/d) | 0.48 (0.16–0.56) | 0.48 (0.16–0.57) | 0.49 (0.07–0.89) | <0.001 |
| Refined grain (serving/d) | 2.0 (1.4–2.3) | 3.8 (3.4–4.6) | 6.0 (4.9–8.9) | <0.001 |
| Fruit juice (serving/d) | 0.10 (0.06–0.23) | 0.134 (0.06–0.34) | 0.18 (0.06–0.54) | <0.001 |
| Other vegetables (serving/d) | 1.14 (0.60–1.92) | 1.22 (0.77–1.97) | 1.41 (0.69–2.53) | <0.001 |
| Potatoes (serving/d) | 0.032 (0.008–0.070) | 0.032 (0.016–0.703) | 0.032 (0.084–0.070) | <0.001 |
| Tea (serving/d) | 1.43 (0.47–2.85) | 1.43 (0.47–2.87) | 0.95 (0.13–2.87) | 0.063 |
| Coffee (serving/d) | 0.008 (0.008–0.032) | 0.008 (0.008–0.032) | 0.008 (0.008–0.032) | 0.002 |
| High fat dairy (serving/d) | 1.08 (0.59–1.47) | 1.12 (0.95–1.64) | 1.10 (0.54–1.79) | 0.678 |
| Green leafy vegetables (serving/d) | 0.23 (0.12–0.45) | 0.30 (0.16–0.56) | 0.33 (0.12–0.68) | <0.001 |

Data represented as mean ±standard deviation (SD), or median (interquartile range (IQR) 25-75) for continues variables and number and percent for categorical variables

* Using ANOVA test for quantitative data and Chi-square test for qualitative data

MET; metabolic equivalent, ELIR, empirical lifestyle index for insulin resistance,

Supplementary Table 4: Study population characteristics based on the tertiles of ELIH score among the Yazd Health Study

|  | T1 (n=1610) | T2 (n=1609) | T3 (n=1611) | P for trend |
| --- | --- | --- | --- | --- |
| **Demographic data** |  |  |  |  |
| Age (year) |  |  |  | <0.001 |
| 20-29 years (%) | 35.4 | 20.5 | 15.6 |  |
| 30-39 years (%) | 22.6 | 25.5 | 23.4 |  |
| 40-49 years (%) | 18.6 | 24.5 | 27.6 |  |
| 50-59 years (%) | 12.6 | 16.9 | 20.3 |  |
| 60-69 years (%) | 10.9 | 12.7 | 13.2 |  |
| Male (%) | 56.8 | 53.3 | 42.8 | <0.001 |
| Body mass index (Kg.m^2^) | 22.6±3.1 | 26.6±3.1 | 30.7±5.2 | <0.001 |
| Physical activity (MET/hour/week) | 19.9±16.9 | 17.2±15.0 | 15.7±13.3 | <0.001 |
| Smoking (yes, %) | 12.5 | 10.9 | 8.9 | 0.005 |
| Menopausal status (yes, %) | 7.6 | 13.7 | 19.8 | <0.001 |
| Marital status (married, %) | 75.9 | 85.8 | 89.2 | <0.001 |
| Education level (diploma and higher, %) | 56.2 | 52.0 | 48.0 | <0.001 |
| Family size (≤ 4 member, %) | 72.9 | 73.1 | 72.7 | 0.962 |
| House acquisition (yes, %) | 73.7 | 76.6 | 81.3 | <0.001 |
| Occupation status (employed, %) | 79.9 | 83.4 | 83.9 | 0.003 |
| Socio economic status (%) |  |  |  | 0.234 |
| Low (%) | 33.0 | 31.0 | 29.8 |  |
| Middle (%) | 43.3 | 45.6 | 46.5 |  |
| High (%) | 23.7 | 23.4 | 23.7 |  |
| Family history of diabetes (%) | 30.7 | 37.1 | 41.2 | <0.001 |
| **Dietary intake** |  |  |  |  |
| Energy intake (Kcal/d) | 2532±984 | 2505 ±942 | 2720 ±961 | <0.001 |
| Carbohydrate (% of energy) | 54.5±7.8 | 53.6±7.6 | 51.1±7.7 | <0.001 |
| Protein (% of energy) | 14.7±3.8 | 15.5±3.7 | 16.3±3.8 | <0.001 |
| Fat (% of energy) | 30.6±6.7 | 30.8±6.9 | 32.5±6.8 | <0.001 |
| Polyunsaturated fatty acids (% of energy) | 8.1±4.8 | 8.2±5.5 | 7.9±4.0 | 0.145 |
| Fiber (g/1000 Kcal) | 9.3±3.8 | 9.0±4.2 | 8.9±4.6 | 0.013 |
| **Dietary components of ELIH** |  |  |  |  |
| Margarine (serving/d) | 0.085 (0.085–0.085) | 0.085 (0.085–0.335) | 0.085 (0.085–0.335) | <0.001 |
| Butter (serving/d) | 0.085 (0.085–0.335) | 0.085 (0.085–0.335) | 0.085 (0.085–0.357) | <0.001 |
| Red meat (serving/d) | 0.17 (0.08–0.27) | 0.27 (0.17–0.43) | 0.45 (0.27–0.74) | <0.001 |
| Fruit juice (serving/d) | 0.16 (0.06–0.36) | 0.11 (0.06–0.36) | 0.12 (0.06–0.43) | 0.002 |
| Coffee (serving/d) | 0.008 (0.008–0.032) | 0.008 (0.008–0.032) | 0.008 (0.008–0.032) | 0.001 |
| Whole fruits (serving/d) | 3.26 (2.62–4.32) | 3.24 (2.54–4.36) | 3.17 (2.58–4.40) | 0.073 |
| High fat dairy (serving/d) | 1.10 (0.77–1.69) | 1.09 (0.67–1.46) | 1.115 (0.68–1.74) | <0.001 |
| Snacks (serving/d) | 0.68 (0.20–1.55) | 0.45 (0.20–1.30) | 0.45 (0.20–0.92) | <0.001 |
| Salad dressing (serving/d) | 0.09 (0.02–0.17) | 0.07 (0.03–0.15) | 0.08 (0.02–0.17) | 0.033 |

Data represented as mean ±standard deviation (SD), or median (interquartile range (IQR) 25-75) for continues variables and number and percent for categorical variables

* Using ANOVA test for quantitative data and Chi-square test for qualitative data

MET; metabolic equivalent, ELIH, empirical lifestyle index for hyperinsulinemia;
